# Analysis of carbon substrates used by *Listeria monocytogene*s during growth in J774A.1 macrophages suggests a bipartite intracellular metabolism

**DOI:** 10.3389/fcimb.2014.00156

**Published:** 2014-11-03

**Authors:** Stephanie Grubmüller, Kristina Schauer, Werner Goebel, Thilo M. Fuchs, Wolfgang Eisenreich

**Affiliations:** ^1^Lehrstuhl für Biochemie, Technische Universität MünchenGarching, Germany; ^2^Abteilung Mikrobiologie, Zentralinstitut für Ernährungs- und Lebensmittelforschung (ZIEL), Technische Universität MünchenFreising, Germany; ^3^Department for Bacteriology, Max von Pettenkofer Institute, Ludwig-Maximilians-UniversitätMünchen, Germany

**Keywords:** bacterial metabolism, bacterial pathogensis, intracellular bacteria, isotopic tracers, isotopologue profiling, *Listeria monocytogenes*

## Abstract

Intracellular bacterial pathogens (IBPs) are dependent on various nutrients provided by the host cells. Different strategies may therefore be necessary to adapt the intracellular metabolism of IBPs to the host cells. The specific carbon sources, the catabolic pathways participating in their degradation, and the biosynthetic performances of IBPs are still poorly understood. In this report, we have exploited the technique of ^13^C-isotopologue profiling to further study the carbon metabolism of *Listeria monocytogenes* by using the EGDe wild-type strain and mutants (defective in the uptake and/or catabolism of various carbon compounds) replicating in J774A.1 macrophages. For this goal, the infected macrophages were cultivated in the presence of [1,2-^13^C_2_]glucose, [U-^13^C_3_]glycerol, [U-^13^C_3_]pyruvate, [U-^13^C_3_]lactate, or a mix of [U-^13^C]amino acids. GC/MS-based isotopologue profiling showed efficient utilization of amino acids, glucose 6-phosphate, glycerol, and (at a low extent) also of lactate but not of pyruvate by the IBPs. Most amino acids imported from the host cells were directly used for bacterial protein biosynthesis and hardly catabolized. However, Asp was *de novo* synthesized by the IBPs and not imported from the host cell. As expected, glycerol was catabolized via the ATP-generating lower part of the glycolytic pathway, but apparently not used for gluconeogenesis. The intermediates generated from glucose 6-phosphate in the upper part of the glycolytic pathway and the pentose phosphate shunt likely serve primarily for anabolic purposes (probably for the biosynthesis of cell wall components and nucleotides). This bipartite bacterial metabolism which involves at least two major carbon substrates—glycerol mainly for energy supply and glucose 6-phosphate mainly for indispensible anabolic performances—may put less nutritional stress on the infected host cells, thereby extending the lifespan of the host cells to the benefit of the IBPs.

## Introduction

*Listeria monocytogenes* is a Gram-positive, food-borne pathogen that can cause systemic infections in immune compromised, pregnant or elder persons (for recent reviews, see Velge and Roche, 2010; Camejo et al., 2011; Fuchs et al., 2012; Mostowy and Cossart, 2012; Cossart and Lebreton, 2014). Typical symptoms of listeriosis are septicaemia, (encephalo)-meningitis, placentitis, and stillbirth. The facultative intracellular pathogen is taken up by professional phagocytes, like macrophages and dendritic cells. It can also actively invade (with the help of the internalins A and/or B) non-phagocytic cells, such as epithelial cells, fibroblasts or endothelial cells (Dussurget et al., 2004; Lecuit, 2005; Hamon et al., 2006). The subsequent escape of the bacteria from the enclosing vacuole depends on listeriolysin and two phospholipases (PlcA and PlcB). Within the cytosol of the host cell, *L. monocytogenes* efficiently multiplies with a generation time of approximately 1 h and spreads into neighboring host cells (Hamon et al., 2012).

The growth of intracellular bacterial pathogens (IBPs) depends on the efficient usage of carbon and nitrogen nutrients from the host. The metabolism of mammalian host cells involves hundreds if not thousands of metabolites that could be used by intracellular bacteria as potential nutrients. The major catabolic reactions of the host cells occur in the cytosol (e.g., glycolysis, pentose-phosphate pathway) or in the mitochondria (e.g., citrate cycle, β-oxidation of fatty acids, glutaminolysis), but metabolites can also be exchanged between these compartments. The anabolic pathways (formation of glucose, amino acids, nucleotides, and fatty acids) mainly take place in the cytosol. Therefore, intracellular bacteria living in the cytosolic compartment of host cells could, in principle, efficiently recruit carbohydrates, amino acids, glycerol, lactate, fatty acids and many other metabolites for their purposes.

Nevertheless, the complex life style of IBPs requires specific metabolic adaptations aimed to optimize survival and proliferation of the pathogen within the different compartments of the host cells. Most features of this complex metabolic interplay between the IBPs and the host cells are still unknown. Even the basic nutrients and their pathways used by the IBPs have not yet been completely elucidated.

Based on the genome sequence, *L. monocytogenes* possesses complete glycolytic and pentose-phosphate pathways (Glaser et al., 2001). Hence, glucose and glucose-6P can in principle be easily catabolized to pyruvate by either of the two pathways. The citrate cycle lacks oxoglutarate dehydrogenase and malate dehydrogenase (Eisenreich et al., 2006). Therefore, and because external Asp can obviously not be imported by *L. monocytogenes*, the formation of oxaloacetate by intracellular *L. monocytogenes* depends fully on the carboxylation of pyruvate catalyzed by pyruvate carboxylase (PycA) (Schär et al., 2010). C_3_- and C_4_-substrates, deriving from glycolytic and TCA cycle intermediates of the host cell could also be taken up by *L. monocytogenes* and may serve as energy source and could be used for gluconeogenesis. Not surprisingly, *L. monocytogenes* therefore multiplies in defined minimal media (Premaratne et al., 1991; Tsai and Hodgson, 2003; Stoll et al., 2008) either containing a PTS-carbohydrate (e.g., glucose, mannose, cellobiose), or glycerol as sole carbon source (Schneebeli and Egli, 2013). Moreover, *L. monocytogenes* is able to utilize glucose 6-phosphate (glucose-6P) which is transported by the phosphate antiporter UhpT. The encoding *uhpT* gene is under the control of PrfA, the central transcriptional activator for most listerial virulence genes (Chico-Calero et al., 2002; de las Heras et al., 2011).

In defined media, *L. monocytogenes* requires Leu, Ile, Val, Met, Cys, and Arg for growth (Premaratne et al., 1991), although all genes encoding the enzymes for the biosynthesis of these amino acids are present on the genome (Glaser et al., 2001) and expressed under suitable conditions (Joseph et al., 2006; Lobel et al., 2012). The requirement for these amino acids may therefore reflect a severe shortage of necessary precursors, especially pyruvate and sulfide, respectively, when growing in minimal media.

The current knowledge about the metabolism of *L. monocytogenes* growing within host cells is still fragmentary and mainly based on ^13^C-isotopologue, transcriptome, and mutant analyses using the established macrophage cell line J774A.1 as host cell (Chatterjee et al., 2006; Joseph et al., 2006; Eylert et al., 2008; Schauer et al., 2010; Donaldson et al., 2011; Lobel et al., 2012). Recently, metabolic studies were also performed with primary murine bone marrow-derived macrophages (BMM) as host cells for infection with *L. monocytogenes* (Gillmaier et al., 2012). Intracellular *L. monocytogenes* replicating in both types of host cells yielded—in the presence of [U-^13^C_6_]glucose—^13^C-isotopologue profiles in the protein-derived amino acids that indicated the usage of glucose-6P and/or glycerol as preferred carbon sources (Eylert et al., 2008; Gillmaier et al., 2012). Indeed, mutants defective in the uptake or catabolism of these carbon substrates displayed reduced growth rates under intracellular conditions. Moreover, genes encoding the transport of glucose-6P (i.e., *uhpT*), as well as the uptake and catabolism of glycerol (i.e., *glpF, glpK*, and *glpD*) were found to be up-regulated under intracellular conditions compared to *L. monocytogenes* growing in a defined medium (Joseph et al., 2006; Lobel et al., 2012).

However, uptake and the possible catabolism of amino acids, glycerol and other C_3_ compounds by intracellular *L. monocytogenes* were not in the focus of the earlier studies. We therefore approached in the present study these questions in considerable detail using ^13^C-glycerol, ^13^C-lactate, ^13^C-pyruvate, or ^13^C-amino acids as tracer substrates in infection assays with *L. monocytogenes* and J774.1 macrophages as host cells. Results from ^13^C-isotopologue profiling underline the important role of glycerol and glucose-6P as major carbon substrates for energy generation and anabolic performances, respectively, and show that most amino acids provided by the host cell are directly used for protein biosynthesis and hardly catabolized.

## Materials and methods

### Materials

[U-^13^C_6_]glucose, [1,2-^13^C_2_]glucose, [U-^13^C_3_]glycerol, [U-^13^C_3_]pyruvate, [U-^13^C_3_]lactate, and a mixture of [U-^13^C]amino acids (ISOGRO ^13^C-Powder Growth Medium) were purchased from Sigma-Aldrich (Steinheim, Germany).

### Bacterial strains and growth conditions

Strains used in this study are listed in Table 1. *Escherichia coli* strain DH5α was used for cloning, and pLSV101 as construction vector for mutagenesis. *E. coli* strains were cultivated in Luria-Bertani (LB) medium at 37°C. *L. monocytogenes* wild type strain EGDe and mutant strains were grown under aerobic conditions in brain heart infusion (BHI) broth or defined minimal medium (MM). If appropriate, MM was supplemented with 10 mM [1,2-^13^C_2_]glucose. When necessary, media were supplemented with erythromycin to final concentrations of 5 μg/ml for *L. monocytogenes* or 300 μg/ml for *E. coli*. To determine growth curves, aliquots were retrieved at regular intervals, and the optical density at 600 nm (OD_600_) was determined using a spectrophotometer. For infection of cells, *L. monocytogenes* strains were grown to the late exponential phase (OD_600_ of 1.0) at 37°C in BHI medium, washed twice with sterile phosphate buffered saline (PBS), re-suspended in 20% (v/v) glycerol in PBS, and stored at −80°C.

**Table 1:** **Strains and plasmids used in this study**.

**Name**	**Characterization**	**References**
EGDe	*L. monocytogenes* Sv 1/2a, wild type, derivative of EGD	
DH5α	*E. coli: deoR endA1 gyrA96 hsdR17*(r_k_-m_k+_) *recA1 relA1 supE44 λthi-1* Δ(*lacZYA*-*argFV169*)	Hanahan, 1983
EGDe ΔC3	In-frame deletion of *glpD* (EGDe Δlmo1293) and *dhaK-1*/*dhaK-2* encoding DHA kinases (EGDe Δlmo0347/lmo0348/Δlmo2695/Δlmo2696)	Mertins, unpublished; Eylert et al., 2008
EGDe ΔC3Δ*uhpT*	EGDeΔC3 with in-frame deletion of *hpt*	Mertins, unpublished; this study
EGDe Δ*uhpT*	In-frame deletion of *hpt*	Chico-Calero et al., 2002
GDe Δ*ldhD*	EGDe with in-frame deletion of *ldh* (lmo0210)	This study
pLSV101	Temperature-sensitive shuttle vector; Em^R^	Joseph et al., 2006
pLSV101-*hpt*del	Deletion plasmid for *uhpT*	Joseph et al., 2006
pLSV101-*ldh*del	Deletion plasmid for *ldh*	This study

### General techniques

Polymerase chain reaction (PCR) amplifications, cloning procedures, isolation of chromosomal DNA and DNA manipulations were carried out according to standard protocols (Sambrook and Russell, 2001). The *Listeria* homepage of the Pasteur Institute (http://genolist.pasteur.fr/ListiList/) and the NCBI database (http://www.ncbi.nlm.nih.gov/) were used for sequence comparison.

### Construction of deletion mutants

Deletion mutants of *L. monocytogenes* EGDe were constructed as described previously (Joseph et al., 2006). Briefly, pLSV101 vector constructs were cloned which carried approximately 0.8–1 kb *L. monocytogenes* EGDe DNA fragments, representing the upstream and downstream sequences of the gene to be deleted. These plasmids were transformed into *L. monocytogenes* EGDe by electroporation, and the bacteria were incubated on erythromycin containing BHI plates at 30°C for 2 day. One single erythromycin-resistant colony was resuspended in 1 ml BHI and 20 μl of this suspension were plated on pre-warmed BHI agar plates containing 5 μg/ml erythromycin, and incubated at 42°C for 2 days. Erythromycin-resistant bacteria growing at 42°C harboring the chromosomally integrated plasmid were selected and subcultivated a few times in BHI without erythromycin at a permissive temperature of 30°C. Erythromycin-sensitive bacteria were screened by PCR for gene deletion from a double-crossover recombination event.

### Cell infection assays

Mouse monocyte macrophages (J774A.1; ACC 170) were received from the German Collection of Microorganisms and Cell Cultures (DSMZ, Braunschweig, Germany) and cultured at 37°C and 5% CO_2_ in RPMI 1640 medium with glutamine (Biochrom KG, Berlin, Germany) and 10% heat-inactivated fetal calf serum (FCS; Perbio Science, Bonn, Germany). Cells were replated twice a week using a dilution rate of 1:3.

### Replication assays

Mouse monocyte macrophages (J774A.1 cells) were seeded at a density of 1 × 10^5^ cells per well in 24-well tissue culture plates (Biochrom) 24 h prior to infection. The cells were washed twice with 0.5 ml of pre-warmed PBS containing 100 mg/ml MgCl_2_ and 100 mg/ml CaSO_4_(PBS-Mg^2+^Ca^2+^), respectively, and infected at a multiplicity of infection (MOI) of 1 bacterium per cell for 45 min. Then, cells were washed with pre-warmed PBS-Mg^2+^Ca^2+^ (*t* = 0 h) before they were overlaid with 0.5 ml of RPMI 1640 containing 50 μg/ml gentamicin and incubated for 1 h at 37°C in the presence of 5% CO_2_. Subsequently, the medium was replaced with fresh medium containing 10 μg/ml gentamicin. At intervals, cells were washed with cold PBS-Mg^2+^Ca^2+^, before the monolayer was lysed with 1 ml of cold Triton X-100 (0.1%). Cell lysates were first vortexed for 30 s, and viable bacterial counts of intracellular bacteria were determined by plating serial dilutions on BHI agar plates.

### Isotopologue profiling of intracellular bacteria

Feeding of living organisms with ^13^C-labeled glucose or other tracers, followed by the determination of the resulting isotopologue patterns in key metabolites (e.g., amino acids) from the bacteria and the host cell fraction, helps to identify substrates and metabolic pathways of intracellular bacteria (Eylert et al., 2008; Gillmaier et al., 2012; Heuner and Eisenreich, 2013; Schunder et al., 2014). Briefly, using this method, ^13^C-labeled substrates (e.g., glucose) are supplied to host cells infected by intracellular bacteria. After uptake of the labeled supplement into the host cell, the tracer is further shuffled into the bacteria where it is utilized for catabolic or anabolic reactions. By these reactions, the label is distributed through the bacterial metabolic network and gives rise to specific isotopologue mixtures in products. Notably, however, with this experimental setting the original labeled precursor could also by first converted into an intermediate or product by the host metabolism that is then incorporated and utilized by the intracellular bacteria. For example, starting from a ^13^C-glucose supplement, labeled pyruvate, lactate, glycerol, alanine and more metabolic products could be generated by the host cell finally serving as substrate(s) for the intracellular bacteria. Nevertheless, the careful comparison of the labeling patterns in multiple bacterial and corresponding host metabolites typically suggests the nature of the preferred bacterial growth substrate and its pathways under intracellular conditions. Frequently, however, these hypotheses must be verified by e.g., corresponding labeling experiments using bacterial mutants defective in the uptake and utilization of the potential substrate.

### Labeling of J774A.1 cells without infection

Cells were seeded in 6 flasks (690 ml/150 cm^2^) and grown to semiconfluence (2 × 10^7^ cells per flask) at 37°C in the presence of 5% CO_2_. Cells were then washed once with pre-warmed PBS-Mg^2+^Ca^2+^, before they were overlaid with RPMI 1640 (Invitrogen, Darmstadt, Germany) without unlabeled glucose, but containing 10 mM [U-^13^C_6_]glucose or 20–40 mM [U-^13^C_3_]glycerol. After incubation with the labeled tracer for 6 h, the cells were washed with 10 ml cold PBS-Mg^2+^Ca^2+^, overlaid per flask with 10 ml PBS containing 50 μg/ml chloramphenicol, 5 μg/ml tetracycline and 20 mM NaN_3_, and shock-frozen for 20 min at −80°C. The frozen suspension was then thawed to room temperature. Cells were harvested by centrifugation at 1000 rpm for 10 min at 4°C. Prior to protein hydrolysis, both supernatant and pelleted cell debris were stored at −80°C.

### Labeling of J774A.1 infected by *L. monocytogenes* EGDe

Bacterial infection was performed in 6 flasks (690 ml/150 cm^2^) per cell line when the cells were semiconfluent (2 × 10^7^ cells per flask). One day prior to the labeling experiment, the colony-forming units per milliliter (cfu/ml) of *L. monocytogenes* EGDe stock solutions were determined in order to exactly adjust the multiplicity of infection. Before infection, the host cells were washed with 10 ml pre-warmed PBS-Mg^2+^Ca^2+^ and then overlaid for 1 h with 20 ml of inoculum per flask composed of FCS-free RPMI 1640 with unlabeled glucose and *L. monocytogenes* (MOI = 25). In order to eliminate extracellular bacteria, the infected cells were then washed with 10 ml of pre-warmed PBS-Mg^2+^Ca^2+^ and overlaid with 20 ml of FCS-free RPMI 1640 with unlabeled glucose containing 50 μg/ml gentamicin and incubated for 15 min at 37°C in the presence of 5% CO_2_. After this time, 20 ml of FCS-free RPMI 1640 containing the ^13^C-tracer source and 50 μg/ml gentamicin to kill non-invaded bacteria were added and incubated for 45 min. Then, the medium was replaced with RPMI 1640 containing 10% FCS, 10 μg/ml gentamicin and one of the ^13^C-tracers specified below. The final concentrations were 10 mM [1,2-^13^C_2_]glucose, 20 mM [U-^13^C_3_]glycerol, 20 mM [U-^13^C_3_]pyruvate, 20 mM [U-^13^C_3_]lactate, or 2g/l of [U-^13^C]amino acid mix (ISOGRO ^13^C-Powder Growth Medium).

After incubation for 5 h at 37°C in the presence of 5% CO_2_, the cells were washed with 10 ml of cold PBS-Mg^2+^Ca^2+^, overlaid per flask with 10 ml of PBS containing 50 μg/ml chloramphenicol, 5 μg/ml tetracycline and 20 mM NaN_3_, and shock-frozen for 20 min at −80°C. The frozen suspension was then thawed to room temperature. In order to remove eukaryotic cell debris, the suspension was centrifuged at 1000 rpm for 10 min at 4°C. For separation of bacteria from soluble eukaryotic protein, the supernatant was centrifuged again at 6000 rpm for 10 min at 4°C. In order to wash the bacterial cells, the pellet was resuspended in 5 ml of RIPA-buffer containing 10 mM Tris (pH = 7.2), 5 mM MgCl_2_, 1% Nonidet P-40, 0.5% deoxycholic acid and 0.1% SDS and centrifuged at 6000 rpm for 10 min at 4°C. Prior to protein hydrolysis, the supernatant containing soluble eukaryotic proteins (in the following “J774A.1 protein fraction”), and the bacterial pellet were stored at −80°C. Owing to the fact that certain amino acids were only labeled in the bacterial fraction, but not in the J774A.1 protein fraction, cross contamination appeared to be <10%.

### Protein hydrolysis and amino acid derivatization

Bacterial cells (approximately 10^9^ cells) or ca. 1 mg of the freeze-dried host protein fraction were hydrolyzed in 0.5 ml of 6 M hydrochloric acid. The mixture was heated at 105°C for 24 h under an inert atmosphere. Under these conditions, protein-derived Gln and Asn were converted into Glu and Asp, respectively. Trp and Cys were destroyed by this treatment. The hydrolyzate was placed on a column of Dowex 50 W × 8 (H^+^ form, 200–400 mesh, 5 × 10 mm). The column was washed twice with 500 μl of water and was developed with 1 ml of 4 M ammonium hydroxide. The eluate was dried under a stream of nitrogen, and the residue was dissolved in 50 μl of dry acetonitrile. A total of 50 μl of N-(tert-butyldimethyl-silyl)-N-methyl-trifluoroacetamide containing 1% tert-butyl-dimethyl-silylchlorid (Sigma) was added. The mixture was kept at 70°C for 30 min. The resulting mixture of tert-butyl-dimethylsilyl derivatives (TBDMS) of amino acids was used for GC/MS analysis without further work-up. The yields of TBDMS-Arg, -Met, -His, -Lys, and -Tyr were low. Therefore, isotopologue data of theses amino acids are only listed when applicable.

### Gas chromatography/mass spectrometry

GC/MS-analysis was performed on a QP2010 Plus Gas Chromatograph/Mass Spectrometer (Shimadzu, Duisburg, Germany) equipped with a fused silica capillary column (Equity TM-5; 30 m × 0.25 mm, 0.25 μm film thickness; SUPELCO, Bellafonte, PA) and a quadrupol detector working with electron impact ionization at 70 eV. One μl of the solution containing TBDMS amino acids was injected in 1:10 split mode at an interface temperature of 260°C and a helium inlet pressure of 70 kPa. The column was developed at 150°C for 3 min and then with a temperature gradient of 10°C min^−1^ to a final temperature of 260°C that was held for 3 min. With a sampling rate of 0.5 s, selected ion monitoring was used. Data were collected using the GC/MS solution software (Shimadzu). All samples were measured three times. ^13^C-Excess and isotopologue abundances were calculated as described before (Lee et al., 1991; Eylert et al., 2008) including: (i) determination of the TBDMS-derivate spectrum of unlabeled amino acids, (ii) determination of mass isotopologue distributions of labeled TBDMS-amino acids, and (iii) correction of ^13^C-incorporation concerning the heavy isotope contributions due to the natural abundances in the TBDMS-moiety and the amino acid atoms.

## Results

### Characterization of *L. monocytogenes* mutants defective in the uptake and catabolism of glycerol, glucose 6-phosphate or lactate

Previous studies (Eylert et al., 2008; Joseph et al., 2008) had shown that glucose-6P and glycerol are important carbon sources for intracellularly replicating *L. monocytogenes*. However, these studies did not rule out the use of amino acids or other C_3_-carbon sources, such as lactate or pyruvate, as additional catabolic carbon sources, nor did these studies show whether glycerol is used for gluconeogenesis. To fill these important information gaps, we made use in the present study of the ^13^C-isotopologue technique to determine the fate of externally added ^13^C-labeled amino acids, ^13^C-glycerol,^13^C-lactate and ^13^C-pyruvate in *L. monocytogenes* EGDe growing within J774A.1 macrophages. In addition to the *L. monocytogenes* EGDe wild-type strain, we included in this study a previously constructed ΔC3 mutant, incapable of utilizing glycerol and dihydroxyacetone due to the loss of glycerol 3-phosphate dehydrogenase and dihydroxyacetone kinases, the Δ*uhpT* mutant, deficient in glucose-6P uptake, and the newly constructed Δ*ldh* mutant, unable to convert lactate into pyruvate due to the lack of lactate dehydrogenase (Table 1). The growth rates of these mutants were similar to that of the wild-type strain when cultured in brain heart infusion medium (BHI) indicating that these mutations did not affect bacterial growth in rich medium (data not shown). These *L. monocytogenes* strains were then infected (at a MOI of 10 bacteria per cell) in J774A.1 cells, cultured in RPMI medium containing 2 mM glutamine and 10 mM glucose or 20 mM glycerol. At the given time points, intracellular bacteria were isolated and counted.

**Figure 1:**
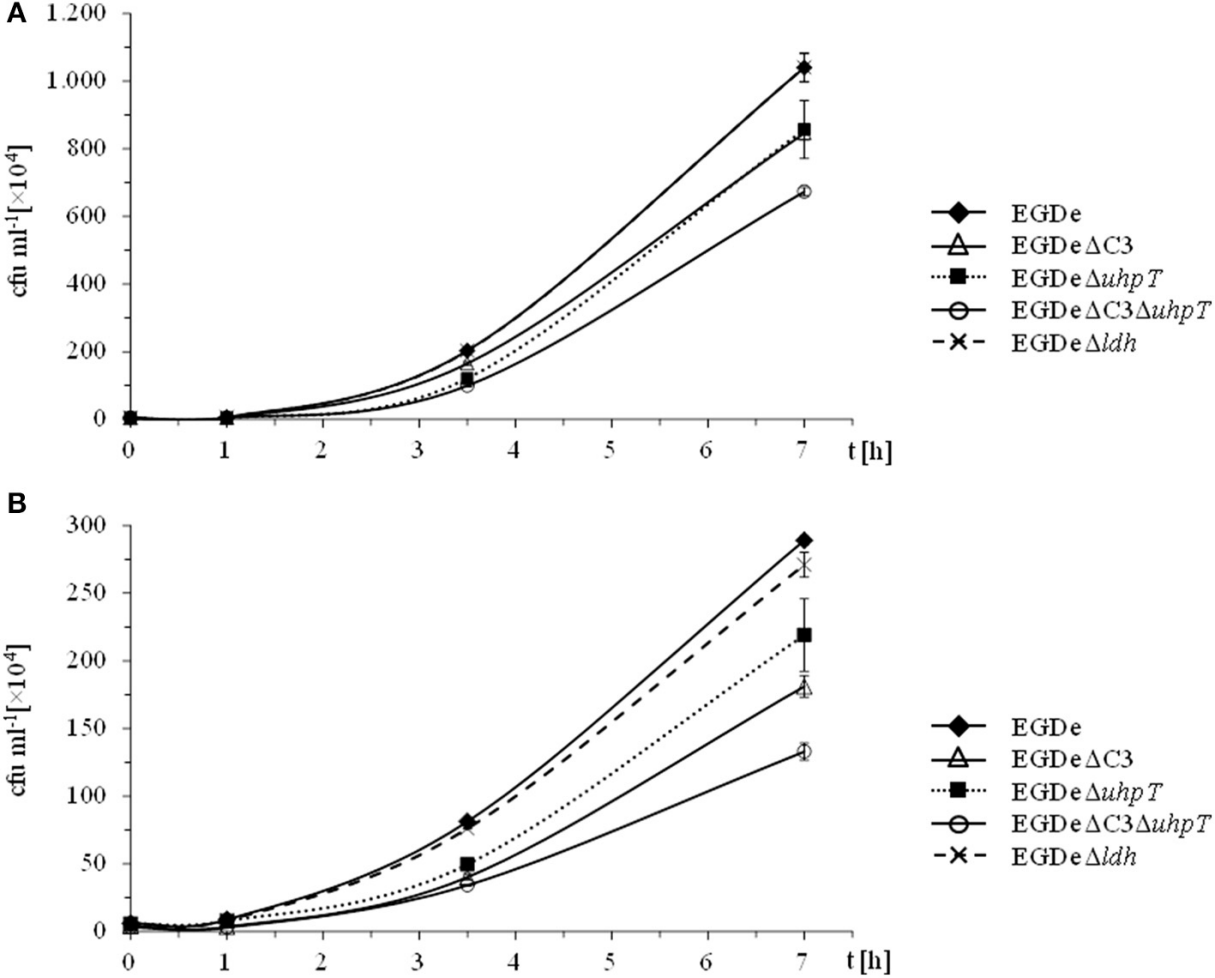
**Intracellular replication behavior of *L. monocytogenes***. *L. monocytogenes* EGDe and its mutants were used to infect J774A.1 macrophages in the presence of 10 mM glucose **(A)** or 20 mM glycerol **(B)**. 45 min after infection, extracellular bacteria were removed by washing with PBS and adding gentamycin. At the indicated intervals, intracellular *L. monocytogenes* were counted by disruption of the monolayer, cell lysis and plating of the supernatant on BHI.

In the presence of 10 mM glucose, growth of the ΔC3, Δ*uhpT*, and ΔC3Δ*uhpT* mutants was inhibited by 20–50% (Figure 1A) which is in line with previous results for the Δ*uhpT* and ΔC3 mutants (Chico-Calero et al., 2002; Joseph et al., 2008). In contrast, growth of the Δ*ldh* mutant was unaffected or even slightly enhanced as compared to the wild-type strain. When glucose was replaced by 20 mM glycerol in the RPMI infection medium, the inhibition of intracellular growth of the ΔC3 mutant was significantly higher (up to 60%) and even the Δ*uhpT* mutant showed reduced growth compared to the wild-type strain (Figure 1B).

These data confirmed the role of glycerol and glucose-6P as major carbon sources for intracellularly growing *L. monocytogenes*, but also showed that, in the absence of these carbon substrates, the listeriae are still able to replicate within the host cells suggesting a switch to alternative carbon sources. Possible candidates are amino acids which can be efficiently taken up by intracellular listeriae from the host cells (Eylert et al., 2008), glucose (or other glycolytic carbohydrates) and some of their catabolic intermediates (e.g., pyruvate or lactate).

### Amino acids imported from the host cells are directly used for listerial protein biosynthesis but hardly catabolized

**Figure 2:**
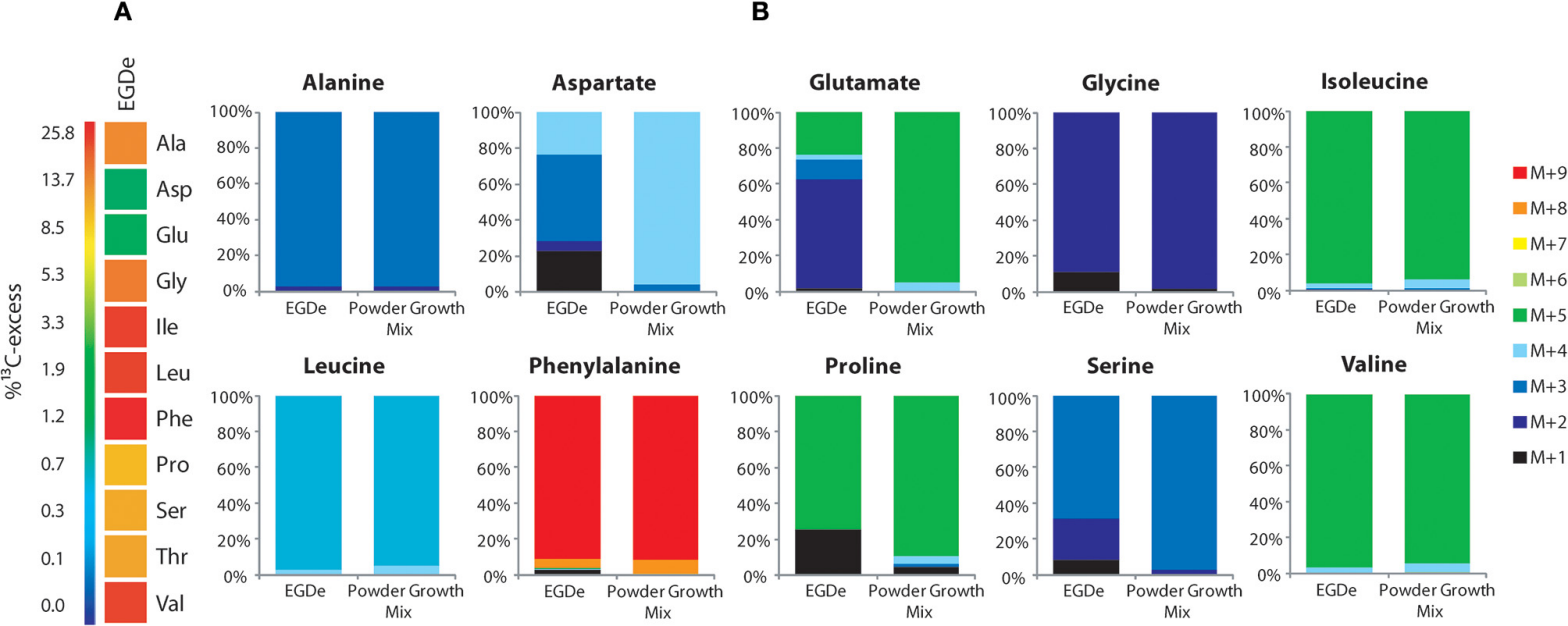
**Isotopologue profiling of protein-derived amino acids from *L. monocytogenes* grown in J774A.1 macrophages labeled with a mix of [U-^13^C]-amino acids. (A)** ^13^C-Excess (in %) of amino acids from *L. monocytogenes* EGDe wild-type. The color map indicates ^13^C excess from 0 to 25% in quasi-logarithmic form. **(B)** Isotopologue profiles in listerial amino acids from the same experiment with EGDe wild-type. For comparison, the isotopologue profiles of the [U-^13^C]-amino acid mix (^13^C-Powder Growth Mix) which was supplied to the infected host cells are also shown. The colored columns indicate the relative fractions (in %) of the ^13^C-isotopologues carrying one to nine ^13^C-atoms (M+1 to M+9). For the numerical values, see Supplemental Material.

To better understand the role of host amino acids in the intracellular metabolism of *L. monocytogenes*, we supplied the infected J774A.1 macrophages (in the presence of unlabeled glucose and Gln) with a mixture of uniformly ^13^C-labeled amino acids (containing all amino acids, except for Asn, Gln, Cys, and Trp) (see Materials and Methods). After 6 h of growth, Ala, Asp, Glu, Gly, Ile, Leu, Phe, Pro, Ser, Thr, and Val were isolated from the acidic hydrolysates of the intracellular bacteria, silylated and analyzed by GC/MS. With the exception of Asp and Glu, the amino acids had acquired substantial amounts of ^13^C-label (about 10% ^13^C-excess, boxes in red or orange) (Figure 2A). Asp and Glu showed only weak ^13^C-enrichments (about 1% ^13^C-excess, boxes in green). Some of the amino acids could in principle also be metabolized by *L. monocytogenes* (e.g., Ala, Asp, Glu, Gly, Ile, Leu, Val, Ser, and Thr). However, all of these amino acids showed, again with the exception of Asp and Glu, the virtually same ^13^C-isotopologue profiles as the respective amino acids in the applied tracer mix (Figure 2B). The unaltered ^13^C-isotopologue patterns of the labeled amino acids indicate that these amino acids were directly incorporated into listerial protein and not significantly catabolized or *de novo* synthesized by the intracellular bacteria.

The fraction of the fully ^13^C-labeled isotopologue in listerial Asp (i.e., M+4 which was introduced by the externally supplied Asp) was very low, and most of the listerial Asp consisted of the ^13^C_3_-Asp isotopologue (Figure 2B). This suggests that intracellular *de novo* synthesis of Asp occurred from ^13^C_3_-oxaloacetate generated by pyruvate carboxlyase-mediated carboxylation of ^13^C_3_-pyruvate (possibly deriving from the supplied ^13^C_3_-Ala). These results were not unexpected as previous studies showed that *L. monocytogenes* is unable to take up external Asp (Schär et al., 2010).

The small ^13^C-labeled amount of listerial Glu carried as a major fraction the ^13^C_2_-Glu isotopologue (Figure 2B) reflecting its *de novo* production from^13^C_2_-oxoglutarate probably deriving from ^13^C_3_-oxaloacetate generated in the TCA cycle. The lack of a larger fraction of uniformly ^13^C-labeled Glu (corresponding to the externally added ^13^C-labeled Glu) is probably due to the large excess of unlabeled Gln in the applied Gln-containing RPMI medium (see Materials and Methods).

Together, the data show that most amino acids imported from the host cell were directly incorporated into bacterial protein and hardly catabolized. Only Asp and (to a minor extent) Glu were synthesized *de novo* by intracellular *L. monocytogenes*, but mainly via intermediates deriving from unlabeled carbon substrates (probably via glycerol and/or glucose-6P) and only to a minor extent from degradation products (e.g., ^13^C_3_-pyruvate) of the supplied ^13^C-labeled amino acids (possibly from ^13^C_3_-Ala).

### Glycerol is efficiently catabolized by intracellular *L. monocytogenes* but not used for gluconeogenesis

Glycerol is an important carbon substrate for intracellular *L. monocytogenes* as shown by several reports (Eylert et al., 2008; Joseph et al., 2008; Lobel et al., 2012). However, none of these previous studies addressed the question whether glycerol is also used for gluconeogenesis. To answer this crucial item, we applied [U-^13^C_3_]glycerol as a tracer in assays with *L. monocytogenes*-infected J774A.1 cells and followed the ^13^C-incorporation into amino acids. In control experiments, we noticed that the complete replacement of glucose by glycerol in the RPMI medium was detrimental to J774A.1 macrophages. But J774A.1 cultures could be kept without damage for several days in RPMI/glutamine medium containing 15 mM glycerol and 2.5 mM glucose. However, infected macrophages cultured in this medium yielded only unlabeled listerial and host cell amino acids possibly due to inhibition of glycerol uptake by the host cells in the presence of glucose. Therefore, we used a modified protocol where the J774A.1 macrophages (pre-grown in RPMI medium with unlabeled glucose for three days) were transferred into fresh RPMI medium containing 20 mM [U-^13^C_3_]glycerol, but no glucose. The J774A.1 cells were then immediately infected with the EGDe wild-type and appropriate mutant strains, and cultivated for 6 h; the infected host cells were then harvested and processed as described.

**Figure 3:**
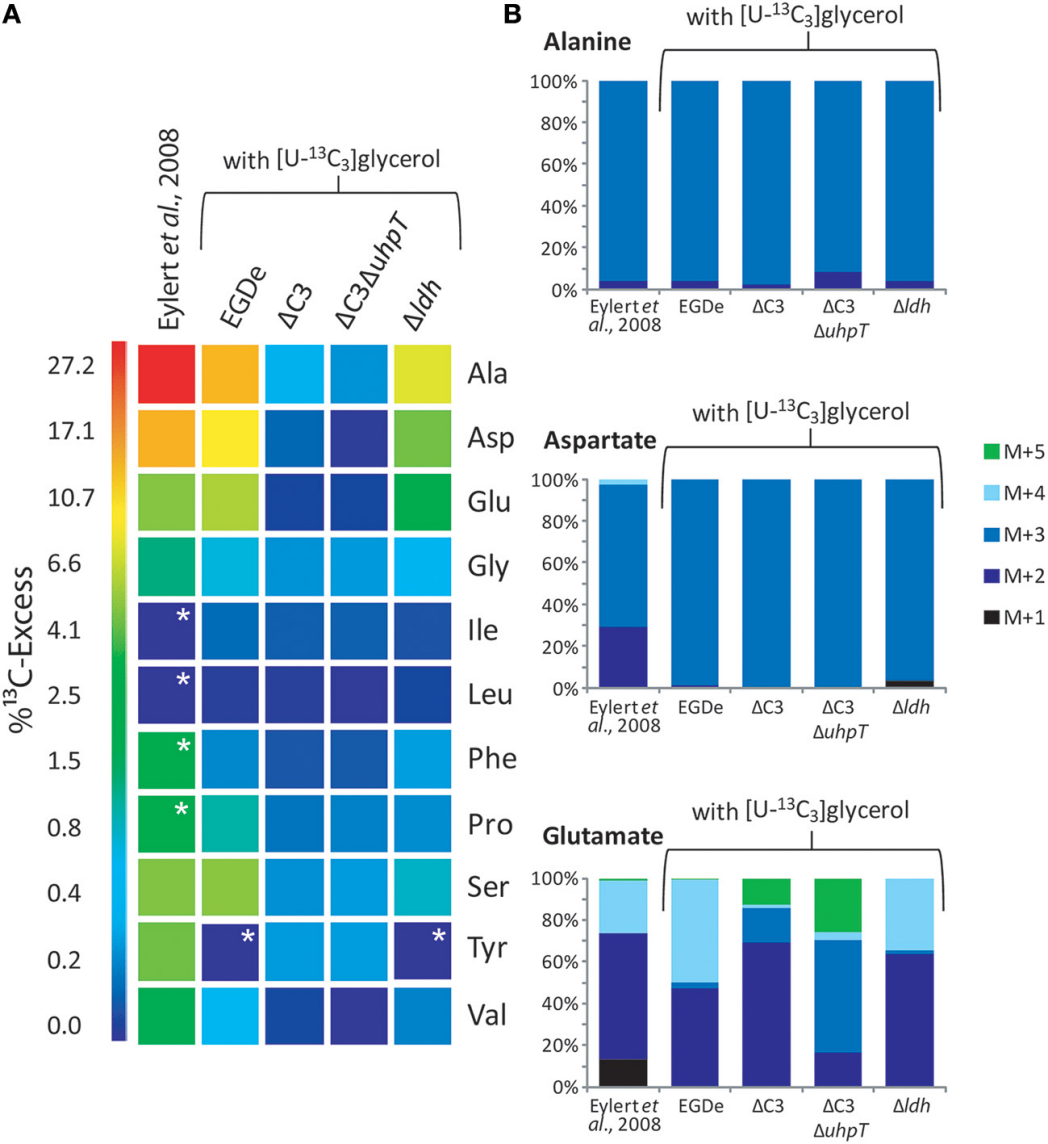
**Analysis of protein-derived amino acids from *L. monocytogenes* grown in J774A.1 macrophages labeled with 10 mM [U-^13^C_6_]glucose (Eylert et al., 2008) or 10 mM [U-^13^C_3_]glycerol. (A)** ^13^C-Excess (in % as a color map) of amino acids from *L. monocytogenes* EGDe. Column 1: from the labeling experiment with [U-^13^C_6_]glucose and EGDe wild-type (Eylert et al., 2008), column 2: from the labeling experiment with [U-^13^C_3_]glycerol and EGDe wild-type (mean value of three replicates), column 3: from the labeling experiment with [U-^13^C_3_]glycerol and the EGDe ΔC3 mutant, column 4: from the labeling experiment with [U-^13^C_3_]glycerol and the EGDe ΔC3Δ*uhpT* mutant, and column 5: from the labeling experiment with [U-^13^C_3_]glycerol and the EGDe Δ*ldH* mutant. Boxes with white asterisks indicate high standard deviations in the measurement of the overall ^13^C-enrichments. **(B)** Isotopologue profiles in listerial Ala, Asp, and Glu from the same experiments. The colored columns indicate the relative fractions (in %) of the ^13^C-isotopologues (M+1 to M+5). For the numerical values, see Supplemental Material.

Under these conditions, ^13^C label deriving from [U-^13^C_3_]glycerol was found in the following listerial amino acids in different quantities: Ala (12% ^13^C-excess) > Asp (8%) > Glu (5%) > Ser (4%) > Pro (1%) > Gly (0.7%) > Val (0.5%) (Figure 3A, column 2). Leu, Ile, Phe, Tyr, and His were detected in unlabeled form. Incorporation of ^13^C into Ala, Asp, Glu, and Ser from the corresponding host cells was observed in much lower amounts (2.5 − 1% ^13^C-excess). This suggests that external glycerol was taken up by the host cells and shuffled into the intracellular listeriae without being catabolized in the host cells in an appreciable amount.

Notably, a similar set of bacterial amino acids acquired ^13^C-label in the infection experiments with [U-^13^C_6_]glucose (Eylert et al., 2008) (Figure 3A, column 1), and the ^13^C-isotopologue patterns were similar in corresponding amino acids from the experiments with [U-^13^C_6_]glucose (Eylert et al., 2008) or [U-^13^C_3_]glycerol, as shown in Figure 3B for Ala, Asp, and Glu. From [U-^13^C_3_]glycerol, Ala was again ^13^C_3_-labeled at high abundance, suggesting that [U-^13^C_3_]glycerol was efficiently converted to [U-^13^C_3_]pyruvate that acted as precursor for ^13^C_3_-alanine. Listerial Asp consisted almost exclusively of the ^13^C_3_-isotopologue obviously via [1,2,3-^13^C_3_]oxaloacetate derived from [U-^13^C_3_]pyruvate and unlabeled CO_2_ and catalyzed by pyruvate carboxylase (PycA). ^13^C-Labeled Glu showed high abundance for ^13^C_2_- and ^13^C_4_-isotopologues, the formation of which can be explained by the assembly of unlabeled or [1,2,3-^13^C_3_]-labeled oxaloacetate with unlabeled or [1,2-^13^C_2_]-labeled acetyl-CoA (obtained from [U-^13^C_3_]pyruvate) in the incomplete TCA cycle resulting in the formation of [4,5-^13^C_2_]-, [2,3-^13^C_2_]-, and [2,3,4,5-^13^C_4_]α-oxoglutarate isotopologues, respectively, which are finally transaminated to the corresponding Glu isotopologues.

In summary, external [U-^13^C_6_]glucose and [U-^13^C_3_]glycerol yielded in the intracellular *L. monocytogenes* a similar set of ^13^C-labeled amino acids with the same ^13^C-isotopologue patterns. In the case of the experiments with [U-^13^C_3_]glycerol, all of the ^13^C-labeled amino acids derive from intermediates generated in the lower part of the glycolytic pathway or the TCA cycle while amino acids requiring intermediates from the pentose phosphate shunt (e.g., His, Phe, and Tyr) were not ^13^C-labeled. These data suggest that both external carbon substrates converge at the level of the same C_3_-glycolytic intermediate (most likely glyceraldehyde 3-phosphate/dihydroxyacetone 3-phosphate) feeding the lower part of the glycolytic pathway and the TCA cycle. These catabolic pathways include all reactions leading to the intermediates required for the observed *de novo* synthesized amino acids and to the generation of ATP by substrate phosphorylation, as well as to NADH/H^+^ necessary for ATP production by oxidative phosphorylation via the electron transfer chain.

Infections of J774A.1 cells with the ΔC3 mutants under these conditions further confirmed the usage of glycerol as major carbon source for intracellular *L. monocytogenes* as the rate of ^13^C-incorporation into Ala, Asp, and Glu dropped in these mutants by a factor of more than 10 compared to the wild-type strain (Figure 3A, columns 3 and 4). This result also showed that ^13^C-labeled glycerol was channeled into the listerial metabolism mainly by glycerol phosphate dehydrogenase which is defective in the ΔC3 mutants.

There still remained, however, a low but reproducible ^13^C-incorporation into Ala, Gly, Pro, and Ser in the ΔC3 mutants (approximately 0.3% ^13^C-enrichments) in the presence of external [U-^13^C_3_]glycerol. These ^13^C-labeled amino acids could be produced in the host cells and transported into the intracellular bacteria. Alternatively, a fraction of [U-^13^C_3_]glycerol could be metabolized in the host cells to other C_3_-components (e.g., [U-^13^C_3_]pyruvate, [U-^13^C_3_]lactate) which are then transported into the intracellular listeriae and subsequently converted into these amino acids.

### Pyruvate and lactate are inefficient substrates for intracellular *L. monocytogenes*

**Figure 4:**
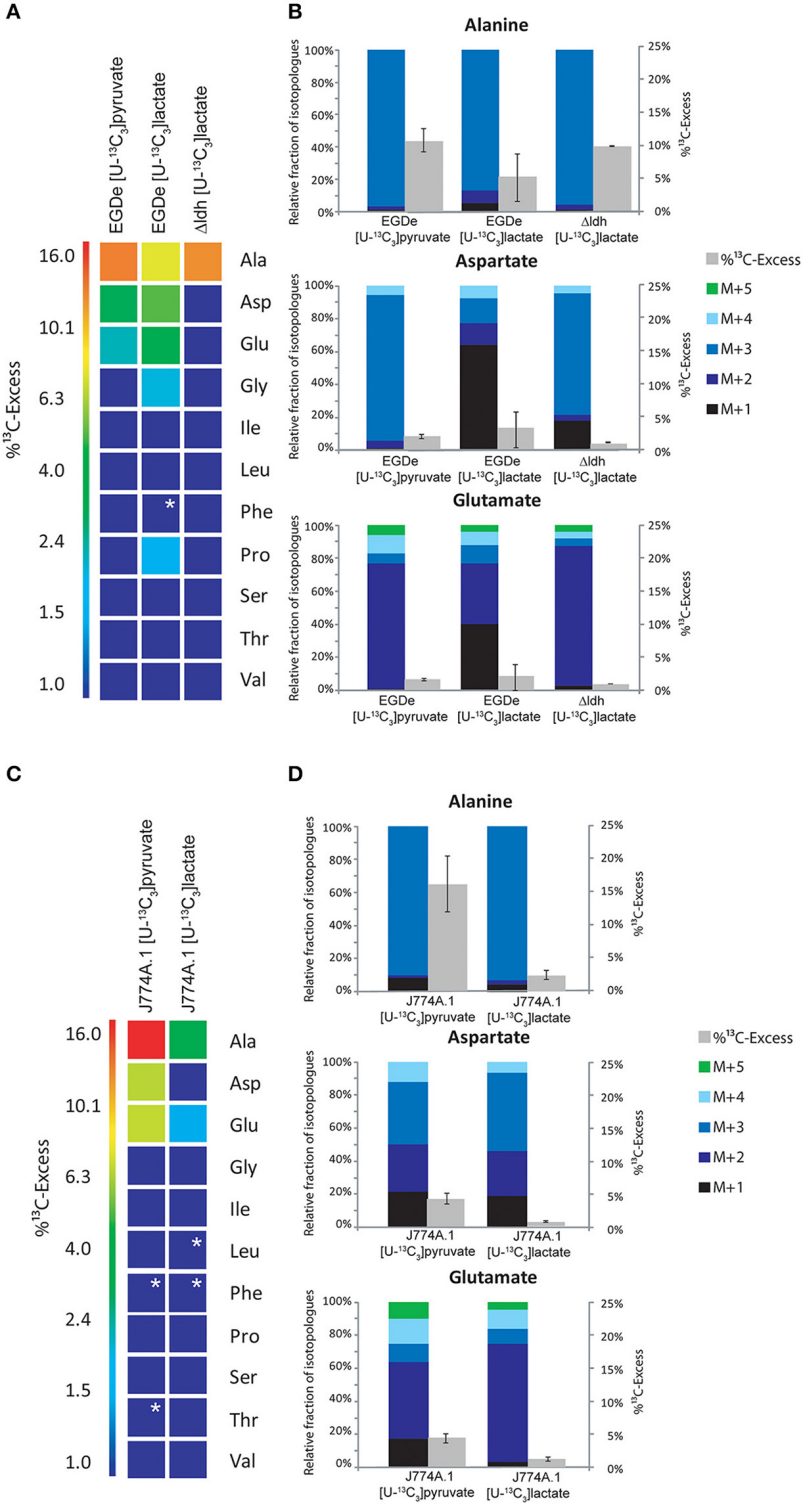
**Analysis of protein-derived amino acids from *L. monocytogenes* grown in J774A.1 macrophages labeled with 20 mM [U-^13^C_3_]pyruvate or 20 mM [U-^13^C_3_]lactate, respectively. (A)** ^13^C-Excess (in %) of amino acids from *L. monocytogenes* wild-type (EGDe) and the Δ*ldh* mutant in the experiment with [U-^13^C_3_]lactate. The color map again indicates ^13^C excess in quasi-logarithmic form in order to also visualize small values. Boxes with white asterisks indicate high standard deviations in the measurement of the overall ^13^C-enrichments. **(B)** Isotopologue profiles in listerial Ala, Asp, and Glu, respectively, from the same experiments. The colored columns indicate the relative fractions (in %) of the ^13^C-isotopologues (M+1 to M+5) in the labeled amino acids (left scales). For comparison, the gray bars indicate the ^13^C-excess values with the standard deviations from three technical replicates (right scales). **(C)**,^13^C-Excess (in %) of amino acids from J774A.1 proteins in the infection experiments with [U-^13^C_3_]pyruvate or [U-^13^C_3_]lactate. **(D)**, Isotopologue profiles of J774A.1 Ala, Asp, and Glu, respectively, from the same experiments. For the numerical values, see Supplemental Material.

In a defined minimal medium, *L. monocytogenes* EGDe is unable to grow in the RPMI medium supplemented with pyruvate or lactate as carbon and energy source (data not shown). To determine whether these two carbon substrates can nevertheless be used by *L. monocytogenes* as supportive carbon substrates under intracellular conditions, *L. monocytogenes* EGDe-infected J774A.1 cells were supplied with 20 mM [U-^13^C_3_]pyruvate or 20 mM [U-^13^C_3_]lactate in addition to equimolar amounts of glucose in the RPMI medium. In the presence of [U-^13^C_3_]pyruvate, Ala, Asp, and Glu from the *L. monocytogenes*-infected J774A.1 macrophages, showed high ^13^C-enrichments ranging from 16% (mostly in form of ^13^C_3_-Ala) to 4% (mostly in form of ^13^C_2_-and ^13^C_3_-Asp, and ^13^C_2_-Glu) (Figures 4C,D). This demonstrated that [U-^13^C_3_]pyruvate was taken up by the macrophages and converted via ^13^C_2_-acetyl-CoA into [^13^C_2_]α-oxoglutarate and [^13^C_2_]oxaloacetate in the TCA cycle and by carboxylation of ^13^C_3_-phosphoenol pyruvate into [^13^C_3_]oxaloacetate.

The amount of ^13^C-label in the same amino acids from the listerial fraction was considerably lower, i.e., Ala (10.5%) > Asp (2%) > Glu (1.5%) (Figures 4A,B). All other amino acids remained unlabeled (<1%) which is different as compared to the infection experiments with uniformly ^13^C-labeled glucose or glycerol. The ^13^C-isotopologue patterns of listerial Asp and Glu were also different than those of the host cells, showing in particular a higher amount of [^13^C_3_]Asp (Figures 4C,D). These data are in line with the assumption that a small amount of [^13^C_3_]pyruvate was either directly taken up by the bacteria or converted by the host cell to other C_3_-compounds, e.g., [^13^C_3_]glycerol, -lactate or -alanine, which were subsequently transported into the intracellular listeriae giving rise to [^13^C_3_]pyruvate/Ala and by PycA-mediated carboxylation to [^13^C_3_]oxaloacetate/Asp.

Indeed, addition of [U-^13^C_3_]lactate to a similar infection assay showed ^13^C-incorporation into bacterial Ala (8.6 − 1.4%), Asp (5.9 − 0.5%), and Glu (4.2 − 0.6%) (Figure 4A). As indicated by the high deviations of the experimental values from several independent experiments (see error bars in Figure 4B), the presence of lactate in the medium might lead to harmful effects for the host cells which can hardly be controlled. We also noticed that the ^13^C-excess values of the bacterial amino acids were higher than those of the corresponding amino acids of the host cells (Figure 4D). Based on this observation, it is likely that lactate provided by the host cells may serve as a direct, but rather inefficient C_3_-substrate for intracellular *L. monocytogenes*. This assumption was further supported by the finding that the Δldh mutant showed ^13^C-label only in Ala (9.5%), but not in any other amino acid (Figure 4A). This ^13^C_3_-Ala was probably generated in the host cell and transported into the intracellular listeriae.

### Glucose 6-phosphate is used by intracellular *L. monocytogenes* mainly for anabolic functions

**Figure 5:**
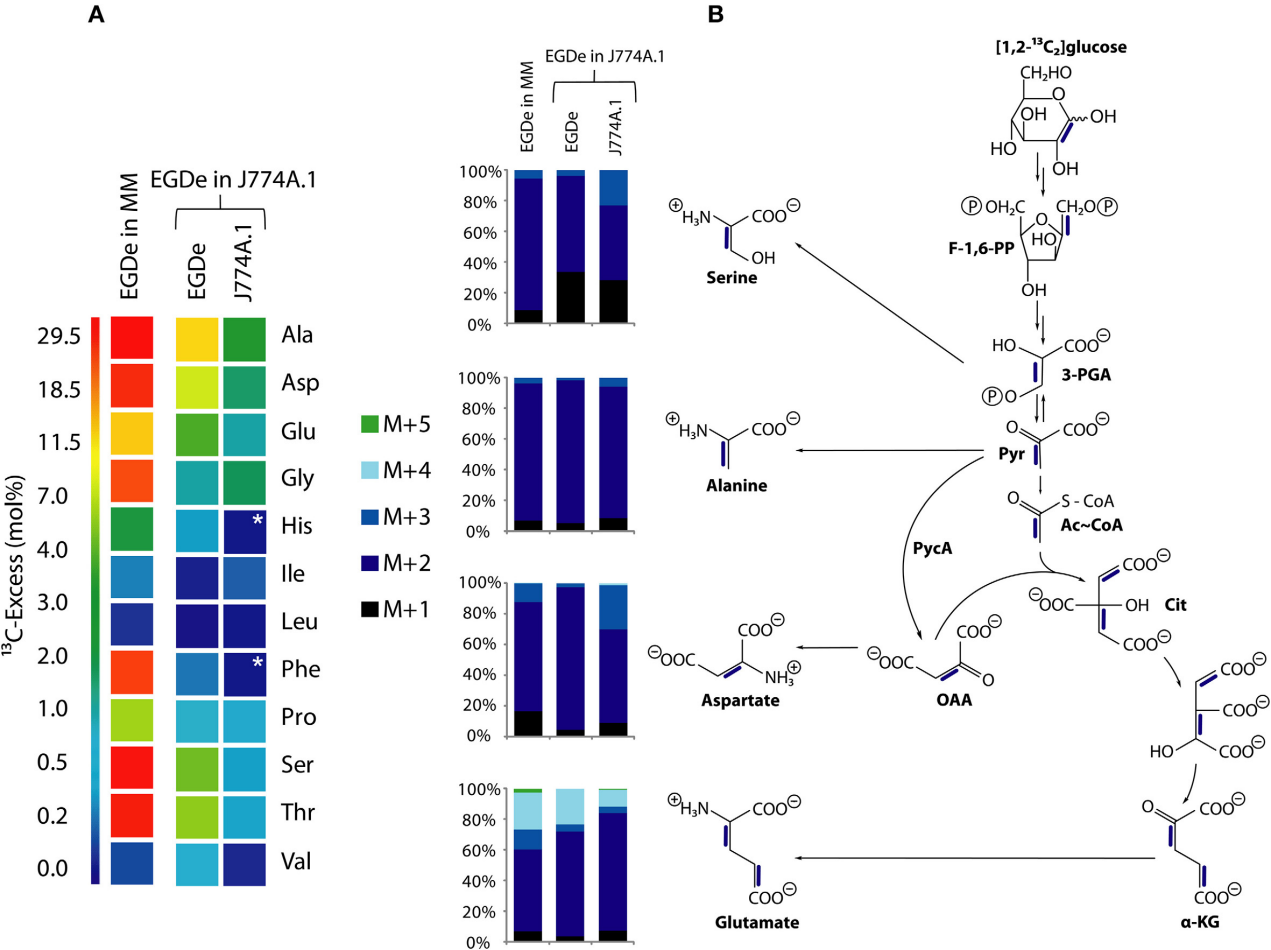
**Analysis of protein-derived amino acids from *L. monocytogenes* EGDe in the experiments with [1,2-^13^C_2_]glucose. (A)** ^13^C-excess (in % as a color map) of *L. monocytogenes* EGDe grown in minimal medium (MM) in the presence of 10 mM [1,2-^13^C_2_]glucose (column 1) of from *L. monocytogenes* EGDe grown in J774A.1 cells in the presence of 10 mM [1,2-^13^C_2_]glucose. Boxes with white asterisks indicate high standard deviations in the measurement of the overall ^13^C-enrichments. Isotopologue profiles in listerial Ser, Ala, Asp, and Glu, respectively, from the same experiments. The colored columns indicate the relative fractions (in %) of the ^13^C-isotopologues (M+1 to M+5) in the labeled amino acids. **(B)** Reaction scheme displaying the conversion of [1,2-^13^C_2_]glucose into the detected isotopologues of Ala, Asp, and Glu. ^13^C-Label is indicated by blue bars. For the numerical values, see Supplemental Material.

Besides glycerol (and, as outlined above, to a minor extent possibly also other C_3_-compounds), glucose-6P was identified in previous studies as an important carbon substrate for intracellularly replicating *L. monocytogenes* (Chico-Calero et al., 2002; Eylert et al., 2008). However, the question remained unanswered whether (i) glucose-6P is catabolized via glycolysis and/or the pentose phosphate pathway, serves as source for energy and the production for intermediates in anabolic pathways or (ii) whether it is mainly required for anabolic functions by its conversion via the pentose phosphate pathway to the sugar components necessary for the biosynthesis of cell wall structures, aromatic amino acids and nucleotides. For this goal, we compared the ^13^C-labeled amino acids of *L. monocytogenes* grown in minimal medium (*in vitro*) and in J774A.1 cells in the presence of [1,2-^13^C_2_]glucose. Although the ^13^C-enrichments in Ala, Asp, and Glu were approximately 3-fold higher from *in vitro* growing *L. monocytogenes* than from intracellular bacteria, the ^13^C-isotopologue patterns of these amino acids were highly similar in both cases (Figure 5A).

More specifically, the MS analysis of the silylated Ala (Ala-260) showed the presence of [^13^C_2_]Ala. To determine the positions of the ^13^C-atoms, a mass fragment (Ala-232) that had lost C-1 during ionization and therefore represents C-2 and C-3 of Ala was analyzed. Ala-232 still showed the presence of two ^13^C-atoms at the same abundance as in Ala-260. It can therefore be concluded that listerial Ala acquired ^13^C-label from [1,2-^13^C_2_]glucose at positions C-2 and C-3. On this basis, pyruvate, the precursor of Ala, was characterized by the [2,3-^13^C_2_]-isotopologue (Figure 5B).

Moreover, the ^13^C-isotopologue pattern in Asp-418 (comprising all carbon atoms of the original amino acids) and Asp-390 (after loss of a carboxylic atom) showed the formation of [2,3-^13^C_2_]oxaloacetate/Asp by pyruvate carboxylase-mediated carboxylation of [2,3-^13^C_2_]pyruvate (Figure 5B). The ^13^C-isotopologue pattern of Glu showing predominantly ^13^C_2_- and ^13^C_4_-species can be explained by the incomplete citrate cycle: [2,3-^13^C_2_]oxaloacetate reacted with unlabeled acetyl-CoA finally yielding [2,3-^13^C_2_]α-ketoglutarate/Glu or with [^13^C_2_]acetyl-CoA (obtained from [2,3-^13^C_2_]pyruvate through decarboxylation by pyruvate dehydrogenase) yielding [2,3,4,5-^13^C_4_]α-ketoglutarate/Glu (Figure 5B). These data are in accord with assumption that the formation of [2,3-^13^C_2_]pyruvate occurs by glycolytic degradation of [1,2-^13^C_2_]glucose, and not via the pentose phosphate pathway, which would result in the formation of [^13^C_1_]- or unlabeled pyruvate.

While the data obtained from *L. monocytogenes* grown in the minimal medium (MM) clearly indicate that the formation of [2,3-^13^C_2_]pyruvate arises from glycolytic degradation of [1,2-^13^C_2_]glucose by the listeriae, this is less obvious in case of the bacteria grown in J774A.1 cells. Here, [2,3-^13^C_2_]pyruvate could be generated by glycolytic degradation of [1,2-^13^C_2_]glucose within the host cell where it is further converted into [2,3-^13^C_2_]glycerol. This compound could be subsequently transported into the intracellular listeriae and further metabolized leading to the observed isotopologues in Ala, Ser, Asp, and Glu. Indeed, the latter explanation appears to be more likely when we compare the other ^13^C-labeled, i.e., *de novo* synthesized amino acids, in MM-grown and in J774A.1 grown listeriae. Thus, MM-grown listeriae showed ^13^C-label (in addition to the above described Ala, Ser, Asp, and Glu) in Gly, Thr, Pro, His, and Phe (Figure 5A). The two latter amino acids require as precursors intermediates from the pentose phosphate pathway, i.e., erythrose-4P and ribose-5P, respectively.

In contrast, *L. monocytogenes* grown within J774A.1 did not show ^13^C-label in these two amino acids. In this case, all ^13^C-labeled amino acids require as precursors for their biosynthesis intermediates from the lower part of glycolysis (Ser, Gly, Ala, Val) or the TCA cycle (Asp, Thr, Glu, Pro) which could be generated from host cell-imported [2,3-^13^C_2_]glycerol.

**Figure 6:**
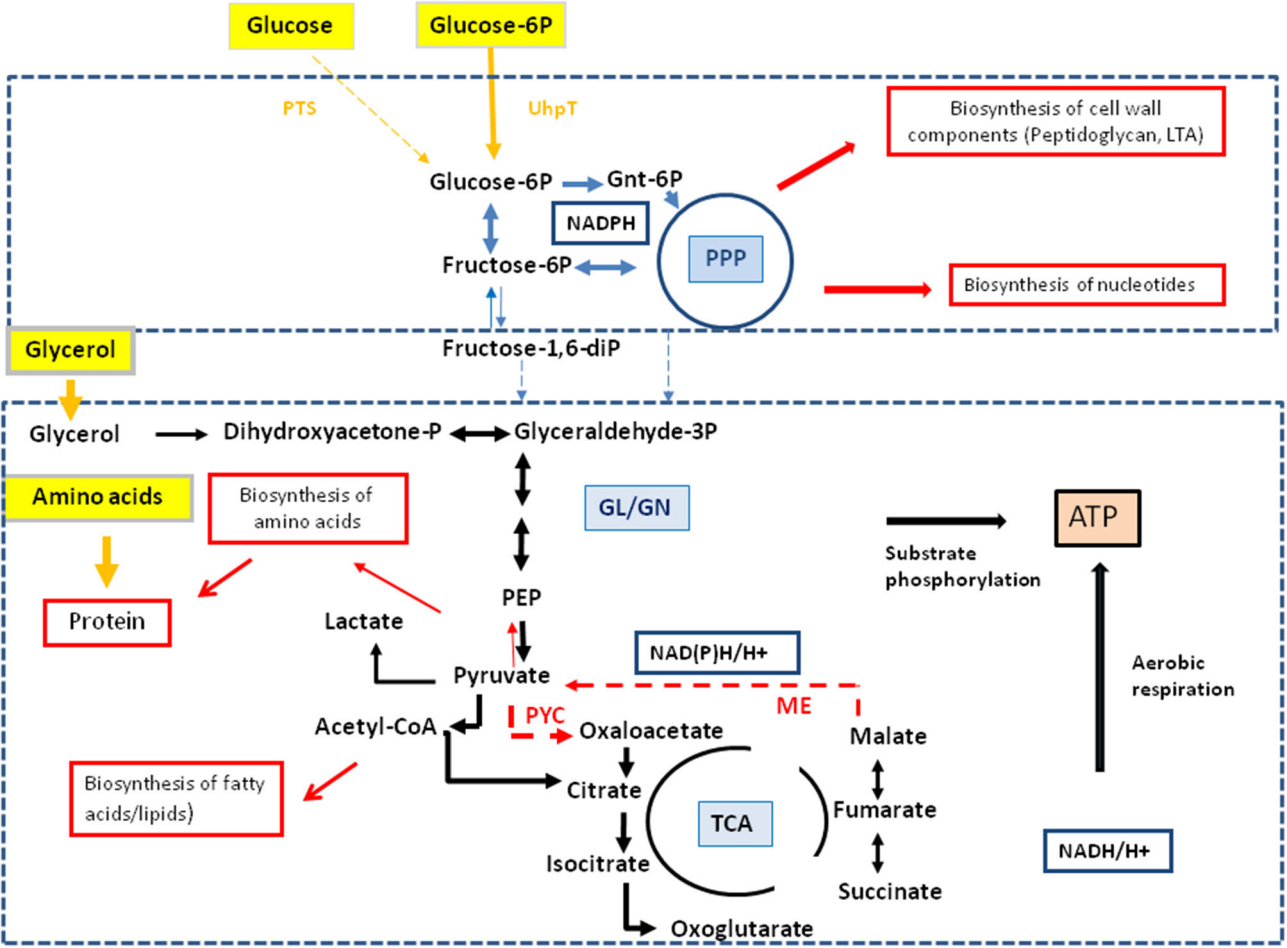
**Model for a bipartite metabolism for intracellular *L. monocytogenes***. Gray-framed yellow boxes and yellow arrows mark (by color intensity) the major carbon compounds taken up by the intracellular listeriae from the host cell. Catabolic reactions fed by glucose-6P and glycerol are indicated by blue and black arrows, respectively. Reactions leading to anabolic performances (in red-framed boxes) are indicated by red arrows. Abbreviations: PPP, pentose phosphate pathway; GL, glycolysis; GN, gluconeogenesis; TCA, tricarboxylic acid pathway; PYC, pyruvate carboxylase; ME, malic enzyme (decarboxylating malate dehydrogenase).

These data indicate that MM-grown and J774A.1-grown *L. monocytogenes* use externally supplied glucose in different ways. While in the MM-grown listeriae glucose feeds the glycolytic pathway and the pentose phosphate shunt, the J774A.1-grown *L. monocytogenes* consume glucose in a dual manner with the participation of the host cell which converts glucose to glycerol and glucose-6P which are subsequently taken up as separate carbon substrates by the intracellular listeriae. Glucose-derived glycerol (generated in the host cell) is used as carbon substrate for the supply of energy and intermediates for the biosynthesis of some amino acids and (probably) fatty acids but not for gluconeogenesis. Vice versa, glucose-6P (also generated in the host cell) may not be catabolized to pyruvate to an appreciable extent, but rather converted in the pentose phosphate shunt into sugar components essential for the biosynthesis of the cell envelope and nucleotides. The aromatic amino acids which require for their biosynthesis erythrose-4P also generated in the pentose phosphate shunt seem to be mainly imported from the host cell. A model for this bipartite intracellular metabolism of *L. monocytogenes* is outlined in Figure 6.

### The carbon metabolism of J774A.1 host cells is differently affected by the wild-type and the ΔC3 and Δ*uhp*T mutant strains of *L. monocytogenes*

This bipartite carbon metabolism carried out by the intracellular listeriae might put less nutrient stress on the host cell thereby extending the lifespan of the host cell—in favor of the intracellular bacteria. Some indirect experimental evidence for this assumption is provided by the different ^13^C-incorporation rates into Ala, Asp, and Glu from the J774A.1 host cells infected with the wild-type, the ΔC3, or the Δ*uhpT* strains. The balanced bipartite metabolism is expected to be disturbed in these two mutants due to their inability of utilize glycerol and glucose-6P, respectively.

**Figure 7:**
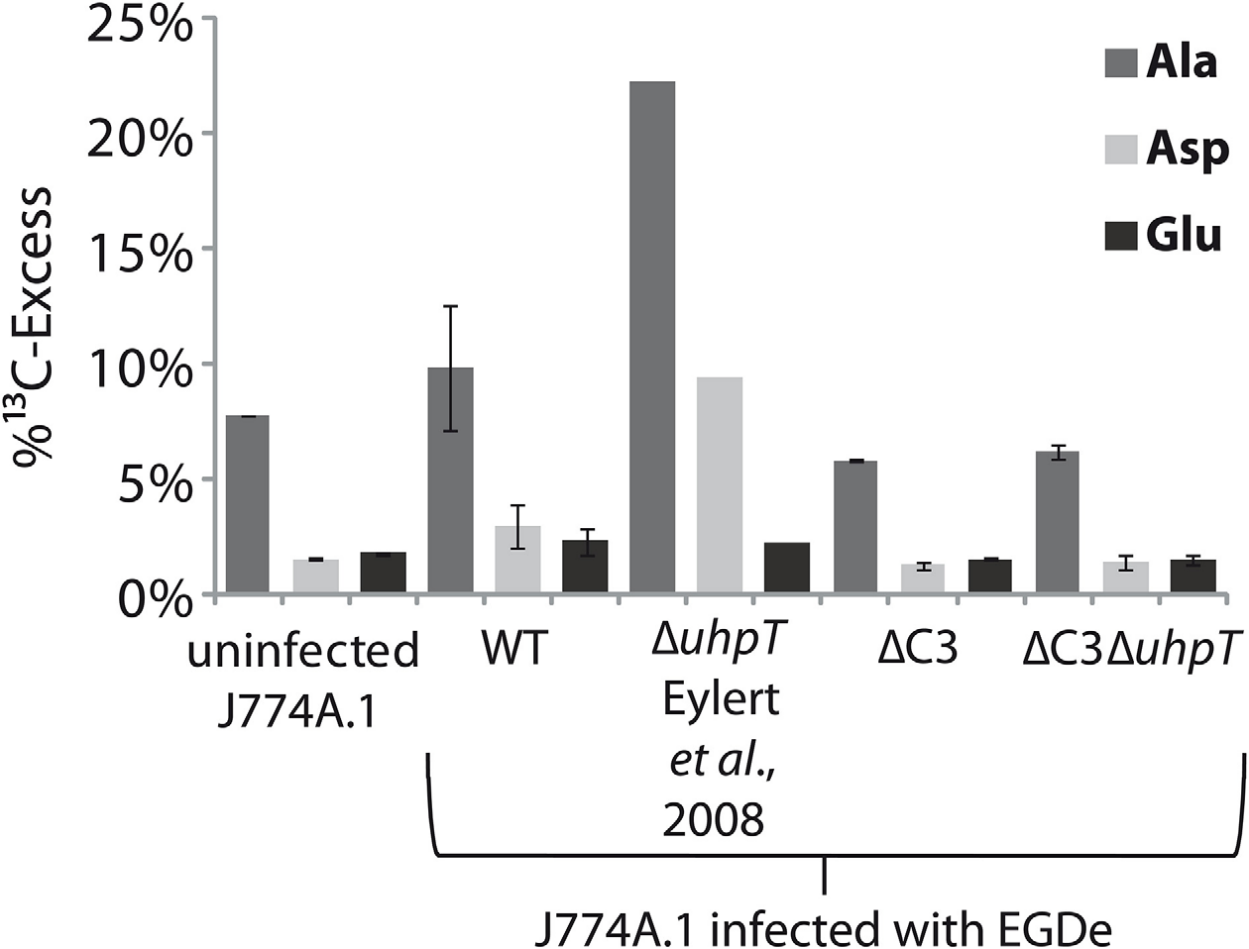
**Analysis of protein-derived amino acids from J774A.1 cells labeled with [U-^13^C_6_]glucose**. The columns indicate ^13^C-excess (in %) of Ala, Asp, and Glu in experiments with uninfected cells, or cells infected *L. monocytogenes* EGDe wild-type, ΔC3, Δ*uhpT*, and ΔC3Δ*uhpT* mutants, respectively.

The J774A.1 host cell line derives from a mouse tumor and expresses c-Myc constitutively (Fan et al., 1993). This leads to enhanced aerobic glycolysis and increased glutaminolysis (Fan et al., 1993; Wise et al., 2008; Dang et al., 2009). In the presence of [U-^13^C_6_]glucose, the most efficiently ^13^C-labeled amino acids of J774A.1 cells were therefore Ala, Glu, and Asp. However, the ^13^C-incorporation rates into these amino acids were slightly but reproducibly increased when the same cells were infected with *L. monocytogenes* EGDe wild-type (WT) (Figure 7) which is in line with earlier observations (Gillmaier et al., 2012). Interestingly, however, ^13^C-incorporation into these amino acids decreased in the J774A.1 cells upon infection with the ΔC3 mutant strains, but increased upon infection with the Δ*uhpT* strain in comparison to the reference experiment with the wild-type strain (Eylert et al., 2008). This effect was less obvious in Glu which is probably caused by the abundant availability of Glu in the J774A.1 host cells due to the enhanced glutaminolysis (Gillmaier et al., 2012). The relatively low ^13^C-enrichment in Glu deriving from of [U-^13^C_6_]glucose (an indication for Glu *de novo* synthesis) is in line with this assumption.

These changes of ^13^C-incorporation into host cell amino acids upon infection by the mutant strains compared to the wild type strain cannot be caused by a different efficiencies of intracellular replication since the number of intracellular wild-type bacteria was similar to that of the mutant strains. The data rather suggest that—as compensation for the defective glycerol or glucose-6P consumption—the resulting increased withdrawal of glucose-6P or glycerol, respectively, from the host cells obviously causes severe changes in the central carbon fluxes of the host cells.

## Discussion

In order to further elucidate the intracellular metabolism of *L. monocytogenes* and in particular for answering the question which carbon substrates provided by the host cells are essential for driving the intracellular listerial metabolism, we performed infection studies with the EGDe wild-type strain and isogenic mutants defective in the transport or catabolism of anticipated carbon substrates and J774A.1 macrophages as host cells. For this goal, we developed experimental protocols for the application of [U-^13^C]amino acids, [U-^13^C_3_]glycerol, [U-^13^C_3_]pyruvate, and [U-^13^C_3_]lactate as tracer carbon substrates to the RPMI culture medium. The efficiency of utilization of these carbon sources and the resulting metabolic fluxes were determined from the ^13^C-isotopologue profiles of the ^13^C-labeled amino acids, a method which we successfully applied before for studying the intracellular metabolism of bacterial pathogens in mammalian cells (Eylert et al., 2008; Götz et al., 2010; Gillmaier et al., 2012).

The results confirm the essential role of glycerol and glucose-6P as carbon substrates for the intracellular listerial metabolism as already suggested by previous studies that were based on transcriptome analyses (Chatterjee et al., 2006; Joseph et al., 2006; Lobel et al., 2012) and on ^13^C-isotopologue profiling studies (Eylert et al., 2008; Gillmaier et al., 2012). The present data also show that other C_3_-carbon substrates, like pyruvate or lactate proposed as possible alternative or supplemental carbon substrates, play only minor roles, if any. It should be noted, however, that even a *L. monocytogenes* ΔC3Δ*uhpT* mutant which is apparently unable to utilize glycerol and glucose-6P can still replicate in the J774A.1 host cells albeit at a reduced rate. This suggests that there are alternative carbon sources which are able to at least partially replace the two major carbon substrates, glycerol and glucose-6P. Possible candidates are glucose, amino acids, glycerol 3-phosphate, or intermediates of the TCA (especially succinate and malate) that could also be provided by the host cells.

The possible participation of glucose and/or mannose in intracellular listerial metabolism is difficult to demonstrate experimentally, since *L. monocytogenes* possess a large number of PEP-dependent phosphotransferase systems (PTS) and even non-PTS transporters for glucose and mannose (Glaser et al., 2001; Stoll and Goebel, 2010; Ake et al., 2011). Deletions of the major glucose/mannose PTS transporters do not seem to affect the intracellular listerial replication. However, in the *pts* deletion mutants, other transporters for these carbohydrates seem to be activated (Stoll and Goebel, 2010).

There is still another reason which makes the participation of glucose or mannose as major carbon source for intracellular listerial metabolism unlikely. The expression of the genes encoding the virulence factors essential for the intracellular listerial life cycle and also the glucose-6P transporter UhpT depends on the central virulence gene activator PrfA (Chico-Calero et al., 2002). However, the activity of PrfA is strongly inhibited when glucose or other glycolytic carbohydrates are used as the major carbon source for listerial growth while PrfA activity is high with glycerol as carbon source (Joseph et al., 2008; Stoll et al., 2008; Götz, unpublished results).

The data presented here also rule out amino acids as important catabolic carbon substrates for the intracellular metabolism of *L. monocytogenes*. An externally added mix of ^13^C-labeled amino acids (containing all amino acids except Trp, Cys, Met, and Arg) is efficiently taken up by the bacteria (with the exception of Asp). However, according to the ^13^C-isotopologue profiles the major glycogenic and ketogenic amino acids are mainly incorporated into listerial protein but hardly catabolized by the intracellular listeriae. This is not surprising for the two most important glycogenic amino acids, Asp and Glu, since Asp cannot be taken up by *L. monocytogenes* (Schär et al., 2010) and the missing oxoglutarate dehydrogenase prevents Asp and Glu degradation in the interrupted listerial TCA cycle (Eisenreich et al., 2006). These results are in line with previous studies (Eylert et al., 2008; Gillmaier et al., 2012) showing limited *de novo* synthesis of most amino acids by intracellular listeriae and import of most amino acids from the host cell in particular of the branched chain and aromatic amino acids. The failure to biosynthesize these amino acids during intracellular growth despite the presence of the biosynthesis capacity on the basis of the genome sequence (Glaser et al., 2001) and transcriptome analyses (Chatterjee et al., 2006; Joseph et al., 2006; Lobel et al., 2012) suggests a shortage of essential catabolic intermediates and/or co-factors required for the biosynthesis of these amino acids under intracellular conditions.

Those amino acids that are *de novo* synthesized by the intracellular listeriae at significant rates derive from intermediates generated in the lower part of the glycolytic pathway (Ser, Gly, Ala) or in the TCA pathway (Asp, Thr, Glu, Pro). These catabolic pathways can be fed by glycerol as major carbon source. Indeed, the nature of ^13^C-labeled amino acids as well as their ^13^C-isotopologue profiles are similar if not identical, irrespective of whether ^13^C-glucose or ^13^C-glycerol were added to the culture medium of the infected host cells. This suggests that ^13^C-glucose is converted in the host cell into ^13^C-glycerol that enters, after being taken up by the intracellular listeriae, the glycolytic pathway at the same position (most likely at the level of glyceraldehyde-3P/dihydroxyacetone-3P) as the externally added ^13^C-glycerol. These glycolytic intermediates are then further catabolized into pyruvate, oxaloacetate and α-ketoglutarate and their down-stream amino acids (see Figure 6), but not used anabolically in gluconeogenesis as indicated by the apparent absence of ^13^C-label in Phe, Tyr and His from exogenous ^13^C-glycerol. *De novo* synthesis of these amino acids would require sugar components, such as erythrose-4P and ribose-5P, respectively, which are generated in the glucose-6P-driven pentose phosphate pathway. Indeed, these amino acids are synthesized and ^13^C-labeled by *L. monocytogenes* growing in an *in vitro* culture medium in the presence of ^13^C-glucose as major carbon source (Eisenreich et al., 2006) as well as by intracellular (also cytosolically) replicating enteroinvasive *E. coli* (Götz et al., 2010). Notably, the latter bacterial pathogen uses mainly glucose as carbon substrate for its intracellular metabolism.

This glycerol-driven metabolism of intracellular listeriae will lead to active PrfA (Stoll et al., 2008) and hence to the expression of the glucose-6P transporter UhpT resulting in the uptake of glucose-6P by the intracellular listeriae. We postulate that this additional carbon substrate may feed the pentose phosphate shunt allowing the generation of the intermediates needed for the biosynthesis of essential cell envelope components and nucleotides. Our presently applied analytical ^13^C-isotopologue approach does not allow the direct detection of these compounds and the experimental prove for this assumption therefore awaits further studies with improved analytical tools.

Together, the data suggest that the intracellular metabolism of *L. monocytogenes* relies on two major carbon substrates: (i) glycerol (generated in the host cell from glucose or other precursors) which is used as carbon substrate for the supply of energy (ATP by substrate-level phosphorylation and aerobic respiration) and of intermediates for the biosynthesis of some amino acids (e.g., Ser, Gly, Ala, Asp, Thr, Glu, and Pro) and (probably) fatty acids, but not for gluconeogenesis, and (ii) glucose-6P (also generated in the host cell from e.g., glucose) which may not be catabolized to pyruvate at significant rates, but rather converted in the pentose phosphate shunt to sugar components essential for the biosynthesis of the cell envelope and nucleotides. The aromatic amino acids which also require for their biosynthesis erythrose-4P, also generated in the pentose phosphate shunt, seem to be mainly imported from the host cell. A model for this “bipartite metabolism” of intracellular *L. monocytogenes* is outlined in Figure 6.

This kind of bipartite metabolism of an IBP could in general have a selective advantage for its survival within the host cell, since it may impose less nutrient stress on the infected host cell than a bacterial metabolism based mainly on the use of glucose. Indeed, *L. monocytogenes* ΔC3 and Δ*uhpT* mutants which are impaired in the balanced utilization of the two carbon substrates seem to impose a greater metabolic burden on the host cells than the wild-type strain as shown by the changes of the isotopologue profiles of host amino acids in infection experiments with *L. monocytogenes* ΔC3 or Δ*uhpT* in comparison to the wild-type strain. Furthermore, enteroinvasive *E. coli* strains which also replicate—similar to *L. monocytogenes*—in the host cells' cytosol, but mainly rely on glucose as a preferred carbon source for the intracellular metabolism (Götz et al., 2010), kill the same host cells much faster than *L. monocytogenes*.

## Author contributions

Wolfgang Eisenreich, Thilo M. Fuchs, and Werner Goebel designed the experiments; Kristina Schauer and Thilo M. Fuchs characterized the mutants, Stephanie Grubmüller and Kristina Schauer performed the labeling experiments, SG performed the GC/MS analysis; Stephanie Grubmüller, Kristina Schauer, Wolfgang Eisenreich, Thilo M. Fuchs, and Werner Goebel analyzed and interpreted the data; and Werner Goebel, Wolfgang Eisenreich, and Thilo M. Fuchs wrote the paper.

### Conflict of interest statement

The authors declare that the research was conducted in the absence of any commercial or financial relationships that could be construed as a potential conflict of interest.
